# Psychedelic Therapy and the Role of Music: A Scoping Review of Quantitative Evidence on Subjective and Objective Outcomes

**DOI:** 10.1002/brb3.71533

**Published:** 2026-06-25

**Authors:** T. Rowe, T. Hurzeler, E. Towers, E. Louie, K. C. Morley

**Affiliations:** ^1^ Sydney Medical School, Faculty of Medicine and Health Discipline of Addiction Medicine, the University of Sydney Sydney New South Wales Australia; ^2^ Edith Collins Centre For Translational Research Royal Prince Alfred Hospital Camperdownc New South Wales Australia

**Keywords:** music, neuroimaging, psychedelic, psychedelic‐assisted therapy

## Abstract

**Purpose:**

Psychedelics have received considerable attention due to their potential in treating psychiatric disorders. The “setting” during psychedelic‐assisted therapy (PAT) is recognized as playing a central role in the experience, during which music features prominently. Although music is theorized as directing and shaping psychedelic sessions, its precise contribution to acute experience and therapeutic outcomes is unclear. This scoping review aimed to map quantitative research on the interplay of psychedelics and music by consolidating existing evidence, identifying gaps, and where possible, reporting on effects of psychedelics and music on subjective (e.g., psychological) and objective (e.g., biological) outcomes.

**Method:**

Following PRISMA (Preferred Reporting Items for Systematic Reviews and Meta‐Analyses) guidelines, relevant papers were identified through electronic databases (MEDLINE, Embase, PsychINFO, Scopus) using terms associated with psychedelic compounds, psychedelic‐assisted therapy, and music. Papers were restricted to quantitative studies published in peer‐reviewed journals investigating human subjects within therapeutic and controlled experimental contexts, focusing on interactions between music and psychedelics.

**Finding:**

A total of 19 papers (total human sample = 330) met inclusion criteria. Psilocybin and LSD were the most studied psychedelic compounds; no studies were found investigating MDMA and music. Characteristics of music conditions across studies have been limited. The findings suggest that music modulates the psychedelic experience through: (1) amplifying and intensifying emotions, (2) recruiting brain networks involved in meaning‐attribution and visual imagery, and (3) increasing overall neural entropy.

**Conclusion:**

Considerable gaps remain in understanding mechanisms of action and how music is delivered to optimize therapeutic response, due in part to methodological inconsistencies and small sample sizes. This review underscores the critical role of music in shaping psychedelic experiences and therapeutic outcomes.

## Graphical abstract

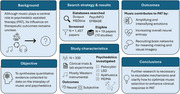

## 1 Introduction

Psychedelic compounds, such as ketamine, 3,4‐methylenedioxymethamphetamine (MDMA), psilocybin, lysergic acid (LSD), and other classic hallucinogens, have seen renewed scientific and clinical interest in recent years. These agents have re‐emerged as potential therapeutic tools whereby preliminary efficacy data exist for a range of psychiatric disorders including depression (Carhart‐Harris et al. 2021), end of life anxiety (Griffiths et al. 2016), post‐traumatic stress disorder (Mitchell et al. 2023), and substance use disorders (Bogenschutz et al. 2022; M. W. Johnson et al. 2014). It has been well documented among pioneering researchers that drug effects can be strongly influenced by the “mindset” of an individual and the environment in which the psychedelic compound is administered, referred to as “set” and “setting” (Leary et al. 1963). Over time, the application of psychedelics evolved from an isolated pharmacological administration toward a more structured therapeutic process with careful consideration of: (i) preparation: supporting participants to develop therapeutic alliance and trust with therapist and psychological readiness prior to dosing; (ii) dosing: guiding participants during the psychedelic dosing session, often with the use of music; and (iii) integration: helping participants during the process of meaning‐making from their experience to maximize therapeutic potential.

Music has long been regarded as a key contextual feature within many Indigenous and ceremonial uses of psychedelics. For example, musical features such as rhythm, tempo, and vocalization are employed within ayahuasca ceremonies observed in the Santo Daime religion to guide emotional experience, evoke autobiographical memory, and shape visual imagery (Efthimiou et al. 2024; Jerotic et al. 2024). Recognition of music's use within ceremonial contexts as a powerful means of shaping the psychedelic experience helped inform early therapeutic uses of psychedelics (Lett and Dyck 2022), where music was increasingly viewed as a means of potentiating drug action (Hoffer 1965), accelerating the release of emotions, and facilitating peak therapeutic experiences (Bonny and Pahnke 1972). Contemporary protocols developed for psychedelic‐assisted therapy (PAT) used across clinical trials have largely inherited this model whereby music remains a near universal component during dosing sessions (Barrett, Preller, and Kaelen 2018; M. Johnson et al. 2008). Despite this, there are currently no formal guidelines to inform optimal music selection for PAT, and music's selection has largely relied on playlists developed by psychedelic therapists using structured approaches corresponding to distinct phases of the unfolding experience (Barrett et al. 2017; Bonny and Pahnke 1972; Messell et al. 2022).

Importantly, a growing body of empirical research examining the role of music in PAT is emerging, explored within recently published narrative (Barrett, Preller, and Kaelen 2018; Efthimiou et al. 2024; Jerotic et al. 2024) and critical reviews (Moskovitz 2025). To date, the only paper to systematically synthesize research on music's use in PAT is O'Callaghan et al. (2020), primarily focusing on qualitative data. Music was found to elicit meaningful emotions and imagery experiences under psychedelics, which participants reported as integral for self‐exploration. However, given the review aimed to understand subjective and qualitative aspects of music during PAT, controlled experimental studies using objective measures (e.g., neuroimaging) were largely omitted. Subsequent research has been published, contributing to a growing body of quantitative research yet to be systematically reviewed.

Research from neuroscience and psychology of music provides a context for understanding how music may shape the psychedelic experience. Indeed, music listening reliably evokes emotion (Juslin 2013) and autobiographical memory (Janata et al. 2007) and modulates large‐scale brain network dynamics (Koelsch 2014). Further, recent models of psychedelic action (see Carhart‐Harris et al. 2014) implicate increased sensitivity to contextual and environmental input; thus, music's effects may be intensified under psychedelics. Currently, no unified theory of music's role in therapeutic uses of psychedelics exists; however, early clinical observations (Bonny and Pahnke 1972) and more recent reviews (Kaelen and Barrett 2018; Vuust et al. 2024) suggest music may play a central role in shaping emotional and perceptual aspects of the psychedelic experience. Despite near universal recognition of music's importance during psychedelic sessions across both Indigenous ceremonial and contemporary clinical settings, understanding music's role during psychedelic experiences and mechanism of action remains poorly understood (Golden et al. 2022), and further efforts are needed to clarify whether it is best conceptualized as acting to scaffold, guide, or contain the experience.

To the authors’ knowledge, there is currently no comprehensive and systematic synthesis of quantitative research investigating the role of music in PAT on influencing clinical outcomes and impacting both subjective (psychological) and objective (biological) processes. The aim of this review is therefore to conduct a comprehensive systematic scoping review with a focus on: (i) consolidating knowledge, identify gaps and characteristics across studies, and, if applicable, (ii) identifying effects of psychedelics and music on subjective (psychological) and objective (e.g., neurobiological) processes.

## 2 Methods

This systematic scoping review was conducted in accordance with the standardized guidelines for PRISMA (Preferred Reporting Items for Systematic Reviews and Meta‐Analyses) guidelines (Liberati et al. 2009).

### 2.1 Search Strategy

A final search was conducted on July 30, 2025, using the following databases: MEDLINE, Embase, PsychINFO, and Scopus. The search strategy incorporated terms only relating to psychedelics, psychedelic assisted therapy, and music. Search terms were identified using a combination of keyword and Medical Subject Headings terms relevant to music psychedelics, and therapy. Search terms for psychedelics were limited to classical serotonergic psychedelics and included psilocybin, LSD, psychedelic, lysergic acid diethylamide, ecstasy, ayahuasca, dimethyltryptamine, hallucinogens, MDMA, and 3, 4‐methylenedioxymethampheatmine. Search terms included for psychedelic‐assisted therapy were psychedelic‐assisted therapy and psychedelic‐assisted therapy. Finally, search terms relating to music were music, music therapy, and music experience. Searches were limited to adults and/or humans to try and eliminate animal studies. Articles were not restricted by year, allowing for the inclusion of papers from early writing on psychedelic therapy. Boolean operators “and” and/or “or” were used alongside subject headings and relevant keywords across databases to ensure comprehensive retrieval of studies.

### 2.2 Paper Selection and Eligibility Criteria

Once all the database searches had been completed and duplicate studies removed, a multi‐stage screening process was performed by two independent authors. Papers were screened in the following order: (i) title and abstract and (ii) full‐length article. Title and abstract were screened to ensure the paper reported on a human population receiving psychedelics while measuring music experience or the effect of music on subjective (e.g., psychological) or objective (e.g., neural) outcomes. In the final stage, all remaining papers had a full‐length text review to ensure eligibility using the following inclusion criteria: participants receiving a psychedelic agent in therapeutic and/or experimental contexts with at least one quantitative measure relating to music experience, assessing (i) the subjective experience of music and or (ii) the impact of music on outcomes relating to psychological, cognitive, or neurological domains. The reference lists of all eligible papers were also manually searched to identify any additional publications.

### 2.3 Data Extraction

The following data were extracted from all eligible papers: author, year of publication, number participants, proportion of males and females, age, clinical group (e.g., healthy controls, depression), setting (experimental, treatment), design (randomized, observational), method (quantitative, qualitative), music characteristics (genre, delivery mode, conditions), outcome (neurobiological including functional connectivity, blood‐oxygenation level dependent [BOLD] signal; physiological including heart rate, blood pressure; psychological including subjective outcomes such as mood, music‐evoked emotions), and relationships or correlations between experience and treatment outcome variables.

## 3 Results

**FIGURE 1:**
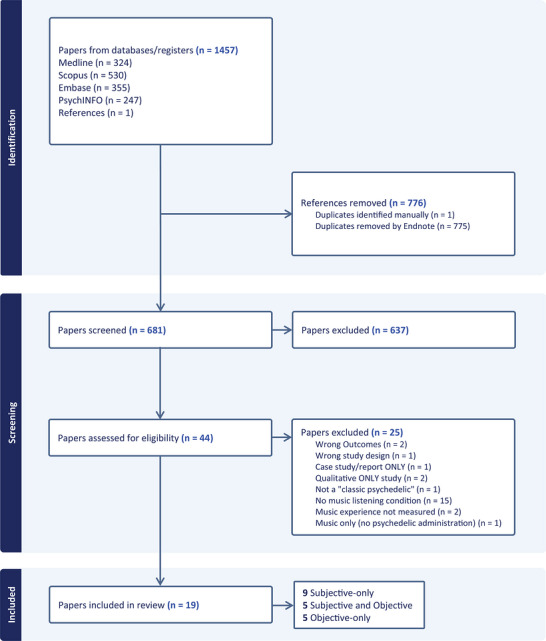
Preferred Reporting Items for Systematic Reviews and Meta‐analyses (PRISMA) flow diagram of the study selection process. Search strategy was completed on July 30, 2025.

**FIGURE 2:**
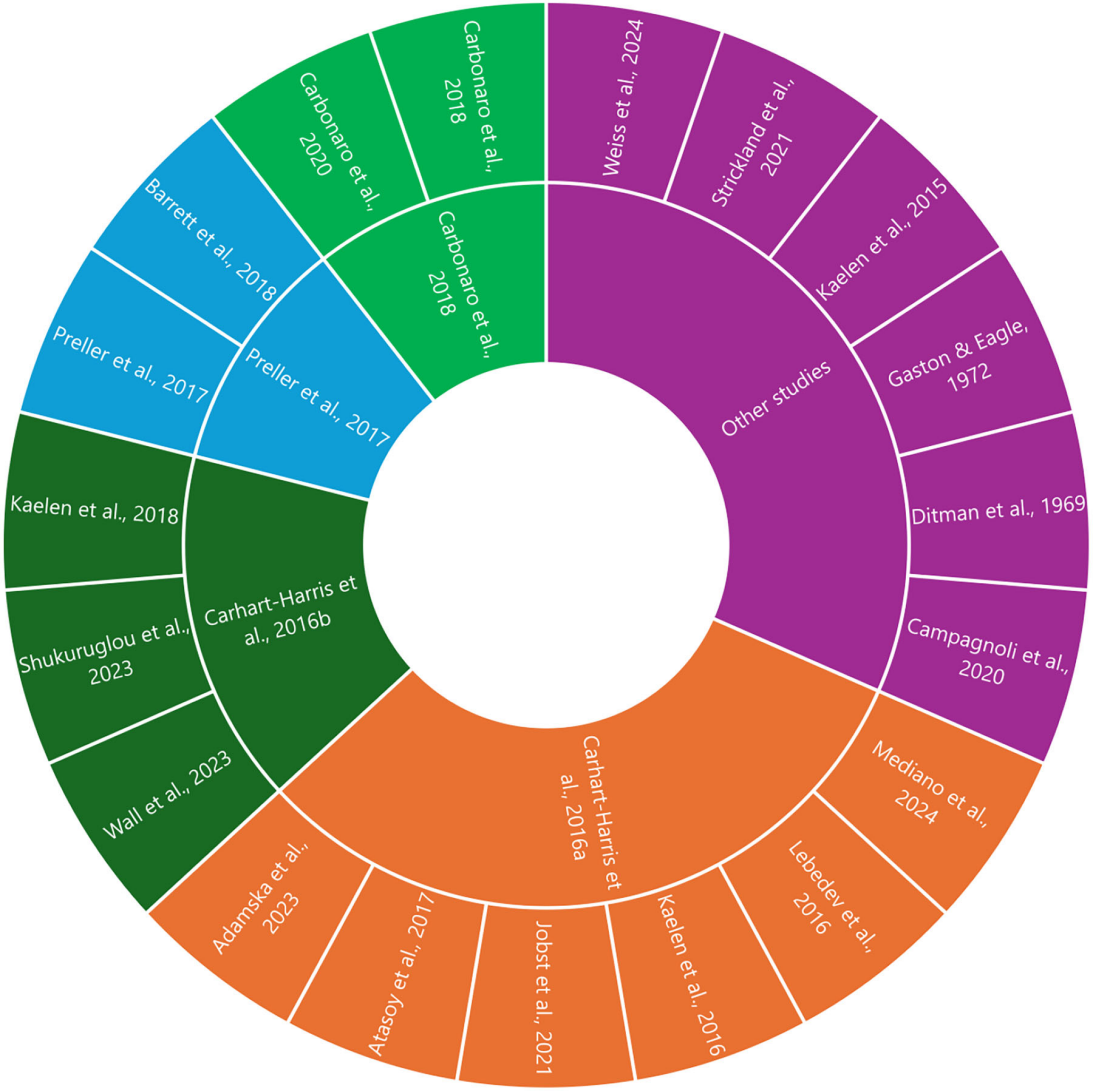
Sunburst chart indicating unique studies (inner‐ring) and individual publications (outer ring).

The initial literature search identified a total of 1457 papers. A total of 776 duplicates were removed along with a further 637 during abstract and title screening. Nineteen papers remained after full‐text screening (Figure 1), representing 10 unique studies (see Figure 2). For clarity, “studies” refer to any of the 10 unique studies, and “papers” refer to any of the 19 included publications. Several papers reported on a subset of participants from their original study dataset due to technical errors encountered during data collection, for example, head movement during functional magnetic resonance imaging (fMRI) acquisition or incomplete recordings. Summaries of characteristics, aims, measures, and outcomes across papers are in Table 1, whereas music listening conditions and music characteristics used across studies are in Table 2. Below, we outline the (i) study characteristics (population, design, intervention, outcomes) and (ii) a summary of the findings.

**TABLE 1:** Summary of included publications.

Authors	Aims	Experimental design	Participants	Psychedelic and dosing	Dosing schedule	Comparisons/conditions	Outcomes and measures	Results
Carhart‐Harris et al. (2016a): LSD experimental study								
Kaelen et al. (2016)	To determine whether listening to music while using LSD modulates the para hippocampal cortex Music = probe/stimulus	Experimental; open‐label; single‐blind; balanced order; placebo‐controlled	*N* = 12 Age: 33 (9) Female 16% Healthy normals	LSD (75 µg, IV) Saline placebo (IV)	Two dosing sessions Interval: at least 14 days	Drug condition: LSD vs. placebo Scan condition: rest (pre‐music), music, rest (post‐music)	Subjective: VAS (simple imagery; complex imagery) Objective: fMRI para hippocampal cortex	LSD increased ratings on simple and complex imagery. No main effects of music or drug × music interaction for subjective ratings but fMRI showed LSD × music interaction with greater parahippocampus coupling to visual and inferior frontal regions, correlating with ratings of complex imagery.
Lebedev et al. (2016)	To determine whether LSD increases the entropy of brain dynamics and that these effects have enduring consequences for personality Music = probe/stimulus	Experimental; open‐label; single‐blind; balanced order; placebo‐controlled	*N* = 19 Age: 30.9 (7.8) Female: 25% Healthy normals Previous experience with psychedelics (NR)	LSD (75 µg, IV) Saline placebo (IV)	Two dosing sessions Interval: at least 14 days	Drug condition: LSD vs. placebo Scan condition: rest (pre‐music), music, rest (post‐music)	Subjective: personality (NEO‐PI‐R); VAS (ego‐dissolution) Objective: fMRI: brain entropy	LSD increased global entropy, associated with changes in NEO‐PI‐R factor *openness*, most pronounced during music‐listening scans. Greater ego‐dissolution during music linked to increased entropy in orbitofrontal and frontoparietal networks, though not significant globally.
Atasoy et al. (2017)	To investigate LSD‐induced changes in brain activity Music = probe/stimulus	Experimental; open‐label; single‐blind; balanced order; placebo‐controlled	*N* = 17 Age: 33 (9); Female: 16% Healthy normals	LSD (75 µg, IV) Saline placebo (IV)	Two dosing sessions Interval: at least 14 days	Drug condition: LSD vs. placebo Scan conditions: eyes closed (resting), eyes closed (with music), eyes open (resting), eyes open (with video)	Objective: fMRI/DTI (energy, power, repertoire, and/or organization of individual harmonic brain states)	LSD expanded the repertoire of active connectome‐harmonic brain states and increased transitions into higher energy (high frequency) states compared to placebo, reflecting greater cortical sensitivity, particularly during music. These effects reduced more rapidly than placebo and were unrelated to subjective imagery ratings.
Jobst et al. (2021)	To analyze the perturbation‐elicited changes in global and local brain activity while listening to music under the influence of LSD Music = probe/stimulus	Experimental; open‐label; single‐blind; balanced order; placebo‐controlled	*N* = 12 Age: 33 (9) Female: 16% Healthy normals	LSD (75 µg, IV) Saline placebo (IV)	Two dosing sessions Interval: at least 14 days	Drug condition: LSD vs. placebo Scan condition: rest (pre‐music), music, rest (post‐music)	Objective: fMRI: dynamical brain changes (and perturbation)	LSD increased global functional connectivity, integration, coupling strength, perturbation latency (PILI), and response variability relative to placebo. Effects were the strongest during music and significant across resting‐state networks.
Adamska and Finc (2023)	To investigate the effect of music, as a part of “setting,” on the brain states dynamics after LSD intake Music = probe/stimulus	Experimental; open‐label; single‐blind; balanced order; placebo‐controlled	*N* = 15 Age: 33(9) Female: 16% Healthy normals	LSD (75 µg, IV) Saline placebo (IV)	Two dosing sessions Interval: at least 14 days	Drug condition: LSD vs. placebo Scan condition: eyes closed (resting), eyes closed (with music), eyes open (resting), eyes open (with video)	Objective: fMRI (brain states dynamics)	LSD and music jointly modulated brain‐state transitions, increasing transitions between task‐positive network states (frontoparietal, dorsal attention, ventral attention). High activity across networks were most affected by the LSD × music interaction.
Mediano et al. (2024)	To investigate how brain entropy is modulated by stimulus manipulation during a psychedelic experience Music = probe/stimulus	Experimental; open‐label; single‐blind; balanced order; placebo‐controlled	*N* = 17 Age: 33 (9) Female: 16% Healthy normals	LSD (75 µg, IV) Saline placebo (IV)	Two dosing sessions Interval: at least 14 days	Drug condition: LSD vs. placebo Scan condition: eyes closed (resting), eyes closed (with music), eyes open (resting), eyes open (with video)	Subjective: VAS (drug intensity, ego dissolution, mood) Objective: MEG: brain entropy across stimulus manipulations	LSD produced significant increases in brain entropy across scan types, with maximal effects in the eyes‐closed scan. Entropy changes correlated with subjective psychedelic intensity, though this relationship was attenuated during video presentation.
Carhart‐Harris et al. (2016b): Psilocybin for depression trial								
Kaelen et al. (2018)	To assess the influence of music on subjective experience of psilocybin with psychological support Music = therapeutic context	Open‐label feasibility trial	*N* = 19 Age^a^: 41.3 (10) Female: NR Diagnosis: major depressive disorder	Psilocybin (10, 25 mg; administration route NR)	Two sessions: weeks 2 and 3 (4‐week feasibility study)	No comparator	Qualitative: Music experience interview Subjective: Psychedelic experience (11D‐ASC), VAS (drug intensity), Depression (QIDS)	Interview‐derived music‐experience scores (liking, resonance, openness) correlated with reductions in depression and components of 11D‐ASC related to mystical experience and insightfulness. Subjective drug intensity rating was unrelated to depression scores.
Shukuroglou et al. (2023)	To examine subjective responses to music before and after psilocybin therapy Music = therapeutic context	Open‐label; feasibility trial	*N* = 19 Age: 43.1 (10.5) Female: 31% Diagnosis: Major Depressive Disorder	Psilocybin (administration route NR) 10 and 25 mg	Two dosing sessions Interval: at least 7 days	Scan condition: pre‐treatment vs. post‐treatment Music condition: music vs. no music	Subjective: VAS (pleasure during scan) Music evoked emotion (GEMS‐21); clinical (SHAP‐S) fMRI (functional connectivity)	Post‐treatment ratings of pleasure in music were increased compared with no‐music and pre‐treatment scans. GEMS scores showed decreased *sadness* and increased *peacefulness* post‐treatment. Greater coupling between nucleus accumbens and default mode network was observed post‐ versus pre‐treatment across music and no‐music. Significant treatment × music interaction demonstrated enhanced coupling during music listening.
Wall et al. (2023)	To examine the effects of psychedelic therapy on the brain's response to music Music = therapeutic context	Open‐label clinical trial	*N* = 19 Age: 41.3(10) Female: NR Diagnosis: major depressive disorder	Psilocybin (10, 25 mg; administration route NR)	Single dosing session for each condition Interval: 7 days	Scan condition: pre‐treatment vs. post‐treatment Music condition: music vs. no music	Objective: fMRI (amplitude of low frequency fluctuations)	Psilocybin therapy increased responses in superior temporal gyrus during music listening. Main effect of scan condition and psilocybin × music interaction, though interaction did not survive multiple comparisons.
Preller et al. 2017: LSD + ketanserin experimental study								
Preller et al. (2017)	To investigate the neuropharmacology of personal relevance processing during psychedelic use Music = probe/stimulus	Double‐blind, randomized, full cross‐over	*N* = 25 (three excluded due to excessive movement) Age: NR Female: NR Healthy normals	LSD (100 µg; oral) Ketanserin (40 mg; oral) Inert placebo (oral)	Single dosing session for each condition Interval: at least 14 days	Drug condition: placebo vs. LSD vs. LSD + KET Music condition: meaningful vs. neutral vs. meaningless	Subjective: VAS (music meaningfulness) Objective: fMRI (personal relevance processing)	LSD increased meaningfulness ratings for meaningless or neutral music versus placebo and LSD + ketanserin. fMRI showed greater activation to meaningless versus meaningful music across frontal, temporal, and limbic regions under LSD, relative to placebo and LSD + ketanserin
Barrett et al. (2018)	To investigate the role of LSD in altering the neural response across various music types Music = probe/stimulus	Double‐blind, randomized, full cross‐over	*N* = 25 (three excluded: excessive movement) Age: 25.68 (3.67) Female: 22% Healthy normals	LSD (100 µg, oral) Ketanserin (40 mg, oral) Inert placebo (oral)	Single dosing session for each condition Interval: at least 14 days	Drug condition: LSD, LSD + ketanserin, placebo Music condition: meaningful, neutral, meaningless	Objective: fMRI (tonal tracking for meaningful and non‐meaningful music)	LSD enhanced neural entrainment to time‐varying tonal changes, with stronger tonal‐tracking observed in frontal, temporal, limbic, and cerebellar regions. Effects were present across drug and music conditions with no significant interaction detected.
Carbonaro et al. (2018): Psilocybin + dextromethorphan experimental study								
Carbonaro et (al. 2018)	To compare subjective, behavioral, and physiological effects of psilocybin and dextromethorphan while controlling for expectancy Music = probe/stimulus	Double‐blind, within‐subjects complete‐crossover	*N* = 20 Age: 28.5 (NR) Female: 55% Healthy normals	Psilocybin (10, 20, 30 mg/70 kg, oral) Dextromethorphan (400 mg/70 kg, oral) Inert placebo (oral)	Five dosing sessions Interval: at least 48 h	Drug condition: psilocybin, dextromethorphan, placebo	Subjective: Psychedelic experience (two items from SOC absorption in music; beauty and significance of music)	Psilocybin demonstrated dose‐related increases relative to placebo and dextromethorphan on items assessing absorption, perceived beauty and significance of music.
Carbonaro et al. (2020)	To examine subjective effects that might account for modest rates of non‐medical psilocybin use Music = probe/stimulus	Double‐blind, within‐subjects complete‐crossover	*N* = 20 Age: 28.5 (NR) Female: 55% Healthy normals	Psilocybin (10, 20, 30 mg/70 kg, oral) Dextromethorphan (400 mg/70 kg, oral) Inert placebo (oral)	Five dosing sessions Interval: at least 48 h	Drug condition: psilocybin, dextromethorphan, placebo	Subjective: Psychedelic experience (single item from SOC related to beauty and significance of music)	Psilocybin increased ratings of music's beauty and significance measured at 1‐week post‐session, with 20–30 mg/70 kg doses remaining higher than placebo and dextromethorphan.
Other studies								
Ditman et al. (1969)	To evaluate the influence of “set and setting” on subjective experience of LSD therapy Music = probe/stimulus	Double‐blind, randomized, between‐subjects	*N* = 99 Age: NR Female: 0% Diagnosis: alcohol use disorder	LSD (200 µg, IV) Methylphenidate (75 mg, IV) Chlordiazepoxide (75 mg, IV)	Single dosing session	Drug condition: LSD vs. methylphenidate vs. chlordiazepoxide	Subjective: Psychedelic experience (DWM card sort test)	LSD increased ratings of perceptual and affective response to music on DWM cart sort test compared with methylphenidate and chlordiazepoxide conditions.
Gaston and Eagle (1970)	To examine the relationship between type of music, music preference, and subjective experience of LSD therapy Music = experimental condition	Experimental; open‐label; partially randomized; between‐subjects	*N* = 59 Age: 46.4 (NR) Female: NR Diagnosis: alcohol use disorder	LSD (500 µg, administration route NR)	Single dosing session	Music condition: no music, familiar music, miscellaneous music, unfamiliar music, familiar music with headphones	Subjective: psychedelic experience (LSD music preference questionnaire; LSD session survey; Check list for LSD experience: researcher rated)	No significant overall differences between music conditions on subjective and objective session ratings; only the familiar‐music‐with‐headphones condition showed pre‐post preference changes. All participants in a subset of those within music conditions reported that music's structure was unchanged and endorsed its inclusion in psychedelic therapy on LSD Survey.
Kaelen et al. (2015)	To determine whether emotional response to music is enhanced under LSD Music = probe/stimulus	Experimental; open‐label; placebo‐controlled	*N* = 10 Age: 34.2 (7.4) Female: 10% Healthy normals Previous psychedelic experience (NR)	LSD (oral) 40 µg (*n* = 1) 50 µg (*n* = 2) 70 µg (*n* = 6) 80 µg (*n* = 1) Inert placebo (oral)	Single dosing session per condition Interval: at least 5 days	Drug condition: placebo vs. LSD	Subjective: VAS (emotional response to music; drug intensity); music evoked emotions (GEMS‐9)	LSD increased ratings of emotional response to music relative to placebo. Emotional response to music positively correlated with subjective drug intensity. LSD increased GEMS‐9 ratings of *wonder, power, transcendence*, and *tenderness* relative to placebo, with emotion response correlating positively with *transcendence*.
Campagnoli et al. (2020)	To investigate the effects of musical stimuli on subjective time during a shamanic ritual in an urban context Music = task/cognitive cue	Experimental; open‐label; double‐blind; balanced order	*N* = 9 Age: 37.7 (NR) Female: 55% Healthy normal	Control ayahuasca (20–60 mL, oral) Experimental ayahuasca (20–60 mL, oral)	Single dosing session per condition (2 doses per session) Interval: variable (up to 20 days)	Drug condition: control ayahuasca vs. experimental ayahuasca vs. no‐drug	Subjective: temporal perception (time‐estimate)	Ayahuasca increased accuracy of subjective time estimation during music listening, with greater effects under higher concentration (experimental ayahuasca) conditions compared to moderate concentration (control ayahuasca) and no‐drug controls.
Strickland et al. (2021)	To test whether music genre played during psychedelic therapy influences subjective and therapeutic outcomes Music = therapeutic context	Open‐label feasibility trial	*N* = 10 Age: 51 (NR) Female: 20% Diagnosis: nicotine‐dependent smokers	Psilocybin (oral) 20 mg/70 kg for first session 30 mg/70 kg for subsequent sessions	Three dosing sessions: weeks 5, 7, and 13 (15‐week treatment period)	Music condition: overtone vs. western classical	Subjective Psychedelic experience (CEQ; MEQ30)	No difference between Western classical and over‐tone‐based music conditions on CEQ and MEQ during dosing sessions 1 and 2. Matching participants to preferred genre for final session did not affect smoking abstinence outcomes.
Weiss et al. (2024) (UK)	To investigate whether acute psychological experiences influence treatment efficacy and clinical response to psilocybin therapy Music = therapeutic context	Double‐blind Randomized controlled trial	N = 59 Age: 41 (NR) Female: 34% Diagnosis: Treatment resistant depression	Psilocybin (oral) 25 mg Escitalopram (oral) 10 mg + psilocybin (oral) 1 mg	Three dosing sessions: week 3 and 6 (6‐week controlled trial)	Treatment: escitalopram therapy vs. psilocybin therapy	Subjective: depression (factor analysis of QIDS, BDI‐IA, HRSD‐17, MADRS); music impact (GEMS‐25 summed score); psychedelic experience (EBI, MEQ30, EDI); VAS (visual imagery)	Music impact moderated treatment response for depression, predicting greater reductions in severity after controlling for subjective psychedelic experience variables.

*Note*: Papers are grouped by study and presented in chronological order.

Abbreviations: 11D‐ASC, 11‐dimensional Altered States of Consciousness Scale; BDI‐1A, Beck Depression Inventory; CEQ, Challenging Experience Questionnaire; DTI, diffusion tensor imaging; EBI, Emotional Breakthrough Inventory; EDI, Ego‐Dissolution Inventory; fMRI, functional magnetic resonance imaging; GEMS, Geneva Emotional Music Scale; HRSD‐17, Hamilton Rating Scale for Depression–17; IV, intravenous; LSD, lysergic acid diethylamide; MEG, magnetoencephalography; MEQ30, Mystical Experience Questionnaire; NEO‐PI‐R, revised five‐factor personality inventory; NR, not reported; QIDS, Quick Inventory of Depressive Symptoms; SHAP‐S, Snaith–Hamilton Pleasure Scale; SOC, States of Consciousness Questionnaire; VAS, Visual Analogue Scale.

^a^Age is reported as mean age (standard deviation) throughout the table.

**TABLE 2:** Music characteristics in studies.

Study	Genre	Playlist information	Listening conditions	Counterbalanced	Music outcome measure
Ditman et al. (1969)	NR	NR	NR	NR	Two items: “I saw music”; music affected my mood much more than usual”
Gaston and Eagle (1970)	Hynms, Rock'n'Roll, Country‐western, jazz, love ballad, folk, march, light classical, heavy classical	Research staff from Topeka Hospital, Kansas	Music was presented by a record player; participants were encouraged to lie down on the bed, put a towel over his eyes, and to relax.	No	LSD Music Preference Questionnaire
Kaelen et al. (2015)	Classical; neo‐classical; ambient; new‐age	Imperial College developed two five‐track playlists (one per session; LSD vs. placebo)	Music delivered via headphones, eyes closed, reclined. Participants could adjust volume. The first 45 min post‐infusion participants listened to ambient music until experimental procedures took place.	Yes	GEMS‐9
Carhart‐Harris et al. (2016a) LSD experiment reported in Kaelen et al. (2016), Lebedev et al. (2016), Atasoy et al. (2017), Jobst et al. (2021), Adamska and Finc (2023), Mediano et al. (2024)	Ambient and classical Indian artists	Imperial College chose two songs for the purpose of the study	Music delivered via headphones, and participants were instructed to close their eyes. Music listening occurred during 7‐min fMRI scans.	Yes	None
Carhart‐Harris et al. (2016b): clinical trial dosing sessions reported in Kaelen et al. (2018)	Ambient; neo‐classical; traditional/ethnic	First dosing session: Imperial College Second dosing session: John's Hopkins	Music played through MRI‐compatible headphones. Participants also wore sound‐attenuating earplugs.	No	Clinician rated music experience: liking, resonance, openness
Carhart‐Harris et al. (2016b): controlled experimental session reported in Shukuroglou et al. (2023) and Wall et al. (2023)	Neo‐classical	Imperial College chose two songs for the purpose of the study	Music played through MRI‐compatible headphones to reduce external noise. Subjects also wore sound‐attenuating earplugs.	Yes	GEMS‐21
Preller et al. (2017) LSD + ketanserin experiment reported in Barret et al. (2018)	Free jazz; traditional folk	Participants and researcher created playlists based on ratings of meaningfulness	fMRI compatible in‐ear headphones and headphones provided to participants	Yes	VAS scales for emotional/meaningful response to music
Carbonaro et al. (2018) psilocybin experiment reported in Carbonaro et al. (2020)	Classical; world music	NR	Music delivered via headphones, eyes closed wearing eyeshades, reclined	NR	Two items: “To what degree are you absorbed in listening to the music?”; “Increase in the beauty and significance of music”
Campagnoli et al. (2020)	Shamanic ceremonial music	University of Sao Paolo researchers developed music	Music delivered via headphones. Participants sat in chairs during music task conditions.	No	None
Strickland et al. (2021)	Overtone; Western classical	Both playlists developed by John's Hopkin's University researchers	Music delivered via headphones and played through room speakers. Participants wore eye mask.	Yes	None
Weiss et al. (2024)	Classical (Indian and Western) and ambient	Imperial College	Music was listened to via headphones, for the entirety of the 4–6 h sessions. Comfortable eyeshades were provided.	No	GEMS‐25 (single measure derived by sum‐scores from all nine factors)

*Note*: Music characteristics across studies are organized in chronological order by year of publication.

Abbreviations: fMRI, functional magnetic resonance imaging; GEMS, Geneva Emotional Music Scale; LSD, lysergic acid diethylamide; NR, not reported; VAS, Visual Analogue Scale.

### 3.1 Study Characteristics

#### 3.1.1 Population

Studies were conducted in the United States (*N* = 4), the United Kingdom (*N* = 4), Switzerland (*N* = 1), and Brazil (*N* = 1), published between 1969 and 2025. Of these, two were conducted during the early era of psychedelic research (Ditman et al. 1969; Gaston and Eagle 1970), whereas the remaining eight were between 2015 and 2025. There were a total of 330 adult participants across studies, comprising cohorts from clinical trials (*N* = 88) and controlled experiments (*N* = 242), of which over half were from the two early studies (*N* = 158). Participants were mostly male and ranged in age from 18 to 80 years, and, where reported, samples were predominantly White and highly educated. Five of the 10 studies recruited subjects from clinical populations: depression (*N* = 2), alcohol use disorder (*N* = 2), and tobacco use disorder (*N* = 1).

#### 3.1.2 Design

Although all studies incorporated psychedelics and music in their analyses, their aims varied considerably. In some, music was a central focus, used to examine its contribution to psychedelic therapy, elucidate mechanisms through which it shapes the psychedelic experience, or explore optimal methods of music delivery to enhance therapeutic outcomes. In others, music played a more peripheral role, serving as a stimulus to probe or evoke subjective phenomena such as meaning or anhedonia or objective outcomes such as modulating brain dynamics. In most studies, participants were exposed to at least one music‐listening condition while under the acute effects of a psychedelic. However, experimental procedures reported in (Shukuroglou et al. 2023) occurred one day after psilocybin administration, and a subset of participants in (Gaston and Eagle 1970) were assigned to a no‐music condition. All study designs incorporated at least one comparator condition, with the exception of open‐label within‐subjects single‐arm clinical trial data reported in Kaelen et al. (2018). However, comparator type and number varied across studies, and no single comparator was common to all experiments. Most studies (*N* = 8) employed within‐subjects repeated‐measures designs, with fewer using between‐subjects (*N* = 2). Study blinding varied, with several using open‐label or unblinded approaches. Carhart‐Harris, Muthukumaraswamy, et al. (2016) was the only study to employ a fully factorial experimental design incorporating both psychedelic versus placebo and music versus no music condition, allowing for the investigation of main and interaction effects of a psychedelic with music.

#### 3.1.3 Intervention

##### 3.1.3.1. Psychedelic Compounds, Doses, and Administration

Relatively few psychedelic compounds were investigated and included LSD, psilocybin, and ayahuasca. Notably, no studies satisfying eligibility criteria investigated MDMA. All three clinical trial studies examined psilocybin administered across two or three therapeutic dosing sessions. The number of psychedelic sessions and doses administered differed across the controlled experimental studies; for example, Carhart‐Harris, Muthukumaraswamy, et al. (2016) administered 75 µg of LSD, whereas Gaston and Eagle (1970) administered 500 µg.

##### 3.1.3.2. Music Conditions

Consistent with established treatment protocols for PAT (M. Johnson et al. 2008), participants in clinical trial studies were presented music using headphones and room speakers, lying down on a couch with eyeshades. In contrast, three of seven experimental studies required subjects to listen to music under neuroimaging scanning procedures (Carhart‐Harris, Bolstridge, et al. 2016; Carhart‐Harris, Muthukumaraswamy, et al. 2016; Preller et al. 2017), during which subjects were provided noise cancelling headphones. Gaston and Eagle (1970) was the only study to deliver music via room speakers rather than headphones. Participants in clinical trials were supported by at least two clinicians during drug administration sessions, whereas subjects could be attended to by researchers and/or clinicians in experimental studies. No papers published from clinical trials reported on quantitative information on participants’ engagement with music during dosing sessions, such as percentage of session spent listening to music or number of participants who requested alterations to music presented. Most studies used fixed, pre‐determined playlists, which were consistent across participants and developed by researchers. Music delivered to subjects varied across studies, though genres of ambient and various classical (e.g., neo‐classical, Western Classical) were most frequently used. Ditman et al. (1969) was the only study not to report on the style of music presented to subjects.

#### 3.1.4 Outcomes

Outcomes across studies were measured using subjective (affective, perceptual, evaluative, task‐based) and objective (neuroimaging) methods, all of which were collected during, immediately following, or within 2 weeks of drug dosing sessions. Kaelen et al. (2015) was the only study to measure physiological outcomes, and although measures were obtained at multiple time points across two study visits, analyses were only conducted at the level of drug condition (e.g., LSD vs. placebo), precluding analysis of music‐specific effects. Subjective measures were administered in all 10 studies and were reported in 15 of the 19 papers. Of the measures specifically assessing music experience, only the Geneva Emotions in Music Scale (GEMS; Zentner et al. 2008) has been formally validated, while others were mainly single or few item scales. Neuroimaging methods were used in three of 10 studies, employing fMRI, magnetoencephalography (MEG), and diffusion tensor imaging (DTI). Broadly, these modalities were used to examine neural outcomes at various levels of functional organization of the brain (e.g., global dynamics to localized regional activity).

### 3.2 Synthesis of Findings Across Papers

Given the heterogeneity of study designs, constructs investigated, and outcome measures used across the papers, the findings were grouped by outcome domain: subjective outcomes only, subjective and objective (neural) outcomes, and objective (neural) outcomes only.

#### 3.2.1 Subjective Outcomes

A total of nine papers reported on subjective outcomes of music under psychedelics. Of these, three reported on clinical trial data and the remaining six reported outcomes from controlled experiments. In an open‐label feasibility trial for nicotine addiction, Strickland et al. (2021) employed a crossover design, randomizing patients to Western classical or overtone‐based music genres during the first two dosing sessions of psilocybin and matched to their preferred genre for the final session to determine how music's delivery (e.g., genre) impacts subjective and therapeutic outcomes. No statistically significant differences were observed across acute‐subjective effect (e.g., mystical experience) or clinical response (abstinence from smoking) outcomes between genres, either during the initial sessions or for the final genre‐matched session. However, authors reported that mystical experience scores tended to be higher in overtone‐based sessions.

Kaelen et al. (2018) and Weiss et al. (2024) examined mechanisms underlying the influence of music on psychedelic therapy response in patients treated for depression, using an open‐label within‐subjects feasibility (Carhart‐Harris, Bolstridge, et al. 2016) and a double‐blind between‐subjects randomized controlled trial (Carhart‐Harris et al. 2021), respectively. Kaelen et al. (2018) used mixed‐methods to quantify music experience variables comprising participants’ openness to, liking of, and resonance with the music experience during psychedelic dosing sessions. The authors reported that the nature of the music experience (openness, liking, and resonance) demonstrated moderate‐to‐strong correlations with mystical‐type and insightful psychedelic experiences (*r* = 0.53–0.70) and with reductions in depression 1 week after psilocybin treatment (*r* = 0.57–0.60). Weiss et al. (2024) demonstrated that greater response on a composite measure of emotional response to music (derived using a sum of scores across all GEMS‐25 emotion subscales, termed “music impact”) was modestly associated with reductions in depressive symptoms (*b* = −0.40), even after controlling for subjective measures of drug intensity and visual imagery.

In Gaston and Eagle (1970), individuals with alcohol dependence participated in an open‐label, partially randomized between‐subjects experiment to determine if ratings on subjective measures of the psychedelic experience were influenced by music's presence, its familiarity, and its mode of delivery. Despite allocating subjects administered with LSD to five between‐subjects music conditions (no music, miscellaneous music, familiar music, familiar music with headphones, unfamiliar music), no differences were found. However, quantitative frequency data from post‐session survey indicated that music was not perceived as distorted, which was of interest to authors given LSD contributed perceptual distortions across sensory domains of taste, smell, and vision. Additionally, all participants allocated to music conditions endorsed music's inclusion in psychedelic therapy.

Several papers reported affective and perceptual responses to music during administration of psychedelics, relative to placebo or active control. In a double‐blind randomized between‐subjects experiment, Ditman et al. (1969) reported increased mood intensity and visual perceptual phenomena during music listening following the administration of LSD compared with both a stimulant (methylphenidate) and a benzodiazepine (chlordiazepoxide). Kaelen et al. (2015), using an open‐label, placebo‐controlled within‐subjects design, reported heightened arousal and enhanced music‐evoked emotions under LSD relative to placebo. In a double‐blind, within‐subjects crossover design, dose‐related increases in aesthetic appreciation and absorption in music under psilocybin compared with dextromethorphan and placebo were reported in Carbonaro et al. (2018) and Carbonaro et al. (2020) both immediately following dosing and at 1‐week follow‐up, respectively. Finally, in an open‐label, balanced‐order, within‐subjects design, participants in Campagnoli et al. (2020) performed a time‐estimation task following 20 brief musical excerpts across varying doses of ayahuasca including a no ayahuasca condition. Time estimations were most accurate under the highest ayahuasca dose relative to other conditions.

#### 3.2.2 Subjective and Objective (Neural) Outcomes

Six papers examined affective, perceptual, and neural responses to music during the administration of psychedelics. Preller et al. (2017) employed a double‐blind, randomized, full cross‐over, within‐subjects design to determine effects of psychedelics and music on personal relevance processing. Participants dosed with LSD, relative to control conditions of placebo and LSD pretreated with 5HT‐2A antagonist ketanserin, attributed greater meaning to music previously rated as personally meaningless or neutral, and increased BOLD signal, via fMRI, was observed in midline cortical and motor regions when listening to meaningless music versus neutral and personally meaningful music.

Shukuroglou et al. (2023) and Wall et al. (2023) reported findings from a subsample of participants from Carhart‐Harris, Bolstridge, et al.’s (2016) open‐label within‐subjects feasibility trial for treatment‐resistant depression. Subjective and neural (fMRI) responses to music were recorded 1‐week before and 1‐day after psilocybin dosing sessions, with both music and no‐music scan conditions at each visit. The findings in Shukuroglou et al. (2023) revealed that music‐evoked sadness decreased and peacefulness increased post‐treatment relative to pre‐treatment. Ratings of pleasure were also higher post‐treatment than pre‐treatment, which were collected at rest (no music) and immediately following music listening. Changes in music‐related pleasure were associated with reductions in anhedonia. Neuroimaging data revealed reduced functional connectivity between nucleus accumbens and regions resembling default mode network (DMN) during music listening, relative to the same comparison conditions. Follow‐up analyses by Wall et al. (2023) reported greater amplitude of low frequency fluctuations (ALFFs) in the bilateral superior temporal cortex and right ventral occipital lobe during post‐treatment music and resting‐state scans, respectively. Voxelwise analysis also revealed relative increases for the music condition in the bilateral superior temporal lobes and supramarginal gyrus, alongside relative decreases in the medial frontal lobes during rest. Additionally, ALFFs within music‐related clusters were significantly correlated with the intensity of subjective effects experienced reported during dosing sessions.

Three publications reported findings derived from the Carhart‐Harris, Muthukumaraswamy, et al. (2016) experiment, a double‐blind, within‐subjects study comparing LSD and placebo under eyes‐closed music and no‐music resting‐state conditions and eyes‐open video and no‐video resting‐state conditions. These publications are presented in chronological order below. Lebedev et al. (2016) investigated changes in brain entropy (measured using sample entropy of BOLD time‐series data) across all conditions. Neuroimaging data (using fMRI and MEG) and subjective self‐report data were collected at various time points, such as baseline, during each scan, or at 2‐week follow‐up. Acute LSD was found to precipitate an increase in global brain entropy across all stimuli conditions, in both sensory and hierarchically higher networks, and across multiple time scales. These shifts in brain entropy were associated with increases in trait openness as measured by the NEO Personality inventory, a relationship which was the greatest during music‐listening scans and when participants reported “ego‐dissolution” during LSD acute dosing period.

Kaelen et al. (2016) investigated the contribution of music to visual‐perceptual phenomena during LSD using the same data. Although music failed to show a demonstrable contribution to self‐reported simple or complex imagery, neuroimaging data indicated that under LSD, music modulated connectivity between parahippocampus (PHC) and both bilateral visual cortex and left inferior frontal gyrus. Further analysis revealed that the strength of coupling between PHC and cortical regions associated with visual imagery correlated with increases in self‐reported complex imagery during LSD (*r* = 0.65). Mediano et al. (2024) reported that LSD produced significant increases in brain entropy across all stimulus conditions. Increases in entropy were the greatest during eyes closed without music condition, followed by eyes closed with music and smaller effects observed across eyes‐open conditions.

#### 3.2.3 Objective (Neural) Outcomes

Four papers reported on objective data collected using various neuroimaging techniques to investigate neural responses to music under psychedelics. Of these, a further three reported on data collected in Carhart‐Harris, Muthukumaraswamy, et al. (2016) and are presented in chronological order below. In Atasoy et al. (2017), DTI and MRI data were analyzed using connectome‐harmonic decomposition to examine individual harmonic brain states in terms of their “power” (strength of activation at a given moment) and “energy” (frequency‐weighted contribution to cortical dynamics). The results indicated that, compared with placebo, LSD expanded the repertoire of active brain states and increased the likelihood of reaching higher energy (higher frequency) states, reflecting heightened cortical sensitivity (particularly during music listening).

Jobst et al. (2021) applied a novel perturbational approach based on a whole‐brain in silico computational model to externally perturb brain regions in silico to investigate relative stability of brain states, or distinct patterns of coordinated brain activity, across drug and music conditions. LSD induced significantly higher Perturbational Integration Latency Index (PILI) values (an index of time taken for brain models to return to dynamical state post‐perturbation) on a global level compared to placebo, indicating LSD may induce shifts away from a stable equilibrium resulting in longer recovery times when returning to equilibrium following perturbation. During music conditions, LSD‐induced increases in functional connectivity were particularly pronounced, with greater differences in PILI values observed within the default mode network compared to the visual network under no‐music conditions. Adamska and Finc (2023) used K‐Means clustering to identify the presence of and dynamic changes between brain states observable across experimental conditions. The results revealed four recurring brain states reflecting sensory, self‐referential, and task‐positive network activity. LSD increased the probability of transitioning between brain states, particularly from a state characterized by self‐referential processing to a task‐positive state. Music appeared to further facilitate LSD‐elicited modulation of brain varying dynamics.

Barrett, Preller, Herdener, et al. (2018) performed follow‐up analyses on data from Preller et al. (2017) by mapping BOLD‐level responses to time‐varying structure changes in music (e.g., rhythm, meter, tonality) using tonal‐tracking (TT), which indexes neural tracking of harmonic structure dynamics and changes in music. LSD demonstrated enhanced neural entrainment or TT bias to musical harmonic structure relative to placebo, with up to seven times more variance in neural activity within frontal and temporal cortical and limbic and cerebellar regions associated with self‐referential processing, emotion, and memory. Similar increases in TT were observed under LSD compared with LSD pretreated with ketanserin.

## 4 Discussion

This comprehensive scoping review synthesized existing literature on music and psychedelics, aiming to map study characteristics (e.g., designs, incorporation of music), consolidate quantitative evidence across subjective (e.g., psychological) and objective (e.g., biological) outcomes, and identify gaps to guide future research. Overall, 19 papers from 10 studies were included, comprising following delivery of music during administration of LSD, psilocybin, and ayahuasca in therapeutic and controlled experimental contexts.

Evidence gathered across studies reporting on subjective response to music under psychedelics revealed alterations across multiple domains. The findings indicate enhanced music‐evoked emotions (Kaelen et al. 2015), increased absorption in and aesthetic appreciation of music (Carbonaro et al. 2018, 2020), intensified mood (Ditman et al. 1969), and modulations in temporal perception, with dose‐related changes in temporal estimation accuracy observed under increasing doses of ayahuasca (Campagnoli et al. 2020), although the implications of these specific effects remain unclear. Together, these findings are broadly consistent with early observations that music may act to shape and direct acute‐psychedelic experiences (Bonny and Pahnke 1972), highlighting the need to clarify how a participant's experience of music during PAT may influence clinical response.

To these aims, two papers contribute preliminary evidence (Kaelen et al. 2018; Weiss et al. 2024). However, it is important to note that given none of the studies included a no‐music condition and current findings rely on indirect associations between music experience and outcomes, direct causal inference regarding music's independent contribution to clinical response in PAT is limited. Nonetheless, the findings from Kaelen et al. (2018) and Weiss et al. (2024) indicate that the quality of a patient's music experience during psychedelic sessions plays a role in amplifying acute‐psychedelic states, exerting influence on treatment outcomes via moderation of acute psychedelic variables such as mystical experience and ego dissolution, both of which are known mediators of clinical response (Ko et al. 2022; Yaden and Griffiths 2021). Qualitative research offer convergent evidence with authors suggesting music's role was that of “hidden therapist” (Kaelen et al. 2018) or, more recently, “sentient actor” (Dwyer et al. 2025), implicated in processes that, on the one hand, serve to deepen and enrich psychedelic states conducive to peak or mystical‐type experiences and, on the other, contribute to distress by evoking unpleasant emotions and imagery that may well be counter therapeutic (Kaelen et al. 2018; O'Callaghan et al. 2020). Given music is positioned as central in guiding emotional processes within traditional and ceremonial entheogenic ritual practices, these findings highlight a pressing need to clarify whether the role of music in PAT is best understood as a guide and director of the experience or simply as a container through which the experience unfolds. Reported findings provide preliminary support for the influence of music in PAT; thus, understanding how music may be delivered to optimize therapeutic response is of importance to researchers and clinicians.

Only two studies (Gaston and Eagle 1970; Strickland et al. 2021) explicitly examined the influence of genre, mode of delivery, or familiarity on subjective experiences. Despite the relevance of this research agenda, the results were largely inconclusive and thus limit conclusions as to how music may best be delivered to optimize therapeutic efficacy in PAT, as discussed in Moskovitz (2025). That said, the findings across both papers indicate that specific characteristics of music (e.g., genre) may play a less prominent role in PAT than previously thought, opening the possibility for psychedelic practitioners working with music to deviate from music generally assumed to be necessary in processes deemed therapeutic (Barrett et al. 2017; Ratkovic et al. 2023). Importantly, the modest evidence gathered coupled with an absence of formal guidelines to inform music selection in PAT stands in contrast to evidence‐based practice guidelines (American Psychological Association Task Force on Evidence‐Based Practice 2006), emphasizing patient preferences, discussed within broader therapeutic literature (Swift et al. 2011) and literature on psychedelics and music (Barrett, Preller, and Kaelen 2018; Jerotic et al. 2024). Given the inherent subjectivity in music preference and variable nature of the psychedelic experience, this represents a significant challenge to PAT practitioners, particularly as group‐based protocols emerge where efforts to balance individual and collective needs in music have produced mixed responses (Lewis et al. 2023). Clearly, more research directly examining the delivery of music in PAT is required to guide these treatment protocols. Elucidating objective biological mechanisms through which music exerts influence under psychedelics could clarify aspects of music that may be important.

Collectively, the findings across papers reporting on objective (neural) outcomes suggest that music functions as a powerful contextual modulator, shaping both localized regional activity and broader large‐scale brain dynamics. At a global level, LSD was shown to increase brain entropy across both sensory and higher order networks, an effect which was deepened during close eyed and music conditions (Lebedev et al. 2016; Mediano et al. 2024). Further, music listening amplified the psychedelic‐associated expansion of brain state repertoire and transitions between different brain states, particularly those involving task positive networks (Adamska and Finc 2023; Atasoy et al. 2017), with computer modelling analysis demonstrating increased time taken to return to base‐level activation following in silico perturbation under LSD, pronounced during music conditions (Jobst et al. 2021). Consistent with theories implicating extrapharmacological or environmental (“setting”) conditions during psychedelic administration, the relationship between brain dynamics and subjective experience demonstrated sensitivity to external stimulation (video vs. music) and attention (eyes open versus closed), whereby visual stimulation reduced the measured entropy–experience relationship (Mediano et al. 2024). These results indicate conditions similar to those recommended in early (Blewett and Chwelos 1959) and contemporary protocols for PAT (M. Johnson et al. 2008), which foster internally focused attention with minimal environmental stimulation (e.g., music listening with eyes closed), may support the relationship between entropic brain states and subjective experience. However, the importance of maintaining the relationship between neural entropy and subjective experience in clinical outcomes requires further clarification, particularly given the role that environmental inputs beyond music may play such as those featured in traditional or group‐based settings and implicated in recently proposed mechanisms of PAT such as nature connectedness (Gandy et al. 2020) or communitas (Kettner et al. 2021).

At a regional level, LSD increased midline cortical and motor region activity, which was associated with heightened personal relevance and meaning‐attribution to music (Preller et al. 2017), as well as enhanced tonal tracking of music in frontal, temporal, limbic, and cerebellar regions (Barrett, Preller, Herdener, et al. 2018). During post‐treatment music, listening psychedelics decreased nucleus accumbens to DMN connectivity (Shukuroglou et al. 2023) and increased ALFFs in bilateral superior temporal and occipital regions (Wall et al. 2023). Finally, music listening under LSD strengthened coupling between the parahippocampus and bilateral visual cortex, as well as between visual and inferior frontal regions, reflecting enhanced emotionally driven visual‐perceptual processing (Kaelen et al. 2016). Thus, while studies directly examining the effect of music versus no music on clinical outcomes during PAT are lacking, objective data indicate that music under psychedelics modulates global brain functioning, affecting regions associated with emotion, meaning‐attribution, pleasure, and visual imagery.

Together, these findings offer preliminary empirical support for music's role in contributing greater flexibility within neurobiological processes and intensified subjective experience. Such effects are consistent with broader research on music in non‐psychedelic contexts (Janata et al. 2007; Juslin 2013; Koelsch 2014), suggesting these effects may be amplified in the presence of a psychedelic. Finally, the results align with mechanistic frameworks such as the entropic brain hypothesis (Carhart‐Harris et al. 2014) and related models such as relaxed beliefs under psychedelics (REBUS; Carhart‐Harris and Friston 2019), which propose that psychedelics relax high‐level predictive constraints and increase the influence of environmental and sensory inputs, such as music, on conscious experience.

### 4.1 Limitations

We observed several limitations in the current literature including methodological limitations. Only a few studies were designed for the current aim a priori, that is, to be a direct investigation of the role of music in subjective or objective outcomes during psychedelic versus placebo exposure. There was only one study that was designed accordingly and could produce a robust interaction effect. Moreover, several of the studies were open label, which could lead to bias with subjective outcomes. Sample sizes were generally small, and where reported, demographic characteristics reflected broader recruitment patterns of recent psychedelic research relying predominantly on White, highly educated participants (Michaels et al. 2018; Vina 2025), limiting generalizability of the findings. Psychedelics investigated across studies were few and, notably, no evidence was gathered on MDMA. Most subjective outcomes relied on single or few‐item scales that were purpose‐built for individual studies and no studies incorporated physiological measures specifically assessing music's effects. There was little consistency in the self‐report measures employed across research settings, which limited direct comparability across studies. Limitations with our review process also exist. Due to the wide scope of the review and limited number of studies, we were limited in the number of methodological variables that could be compared quantitatively.

### 4.2 Conclusion and Future Directions

This review provides the most comprehensive synthesis to date of the role of music in psychedelic literature and the impact on subjective and neurobiological outcomes. Objective biological data to date, although still preliminary in nature, point toward a key role of music in enhancing neurobiological response to psychedelics. Data regarding the subjective experience also provide preliminary support regarding the influence of music in PAT. However, little data exist from rigorously designed studies to guide our understanding with regard to the delivery of music. Further research is required to explore the following: adherence to Reporting of Setting in Psychedelic Clinical Trials guidelines (ReSPCT; Pronovost‐Morgan et al. 2025) for music within dosing sessions, training needs for therapists working with music, the potential of music during integration and impact on outcomes, pre‐determined “safety” music employed during intensely distressing experiences, and clarity on measurement instruments to be used consistently across research settings.

## Author Contributions

**T. Rowe**: conceptualization, methodology, validation, writing – original draft. **T. Hurzeler**: methodology, validation, visualization, writing – review and editing, supervision. **E. Towers**: writing – review and editing, validation. **E. Louie**: validation, writing – review and editing, methodology. **K. C. Morley**: supervision, conceptualization, investigation, funding acquisition, writing – review and editing.

## Funding

The authors have nothing to report.

## Ethics Statement

No ethical was required for this review.

## Conflicts of Interest

The authors declare no conflicts of interest.

## Data Availability

Data sharing not applicable to this article as no datasets were generated or analyzed during the current study.
